# Mumps Outbreaks at Four Universities — Indiana, 2016

**DOI:** 10.15585/mmwr.mm6729a1

**Published:** 2018-07-27

**Authors:** Mugdha Golwalkar, Brian Pope, Jill Stauffer, Ali Snively, Nakia Clemmons

**Affiliations:** ^1^Indiana State Department of Health; ^2^Division of Viral Diseases, National Center for Immunization and Respiratory Diseases, CDC.

From February to April 2016, the Indiana State Department of Health (ISDH) confirmed mumps outbreaks at four universities (three public and one private). All universities were located within 65 miles of Indianapolis; however, epidemiologic links among outbreaks were limited. ISDH and local health departments investigated the outbreaks and initiated control measures at all universities. A protocol describing recommended testing for mumps, testing priorities during the outbreak, and a preauthorization process for submitting specimens to the ISDH Laboratory (ISDHL) was developed and disseminated to providers and public health partners (*1*). Outbreaks at each university were declared over after two incubation periods* elapsed without identified cases; the last outbreak ended September 10, 2016. Among the 281 confirmed and probable cases identified, 216 (76.9%) persons had documentation of presumptive evidence of immunity^†^ (*2*). At some universities, documentation of receipt of 2 doses of measles, mumps, rubella vaccine (MMR), which is a criterion for evidence of immunity, was not available and required substantial personnel time to verify. Implementation of policies for excluding susceptible persons from classes and other group settings was also difficult. The laboratory testing protocol increased the percentage of specimens testing positive and improved case detection. Outbreak-specific laboratory testing guidance on specimen collection for mumps confirmation and standardized vaccination documentation in highly vaccinated settings could aid outbreak management. Evaluation of exclusion policies might also be necessary. In 2018, the Advisory Committee on Immunization Practices (ACIP) published a recommendation that persons previously vaccinated with 2 doses of MMR who are determined by public health authorities to be part of a group at increased risk for infection during a mumps outbreak receive a third dose of MMR (*3*).

## Investigation and Results

On January 20, 2016, a student with unknown mumps vaccination history was evaluated at university A’s student health center for parotid swelling. The student reported a possible mumps exposure at a university outside Indiana, where a large mumps outbreak was occurring. Mumps immunoglobulin M (IgM) testing was negative, but continuing parotitis motivated the university to request reverse transcription–polymerase chain reaction (RT-PCR) testing at ISDHL 2 days later, and results were positive for mumps. By February 17, 2016, two additional cases at university A were confirmed by RT-PCR. On January 25, a fully vaccinated student was evaluated at university B’s student health center with parotid swelling, headache, and fever. Mumps was suspected and reported to ISDH; however, laboratory testing was not conducted. On February 12, three additional mumps cases with epidemiologic links to the index case were confirmed by RT-PCR at university B. On March 11, three cases were confirmed by RT-PCR at university C, with no epidemiologic links among the patients or to any outside case. On April 2, three cases (one confirmed by RT-PCR and two epidemiologically linked) were identified at university D; all patients reported possible exposures to mumps during a spring break trip to Florida 2 weeks before symptom onset. Additional mumps cases occurred in all four universities and in the surrounding community, with the last onset date among university-affiliated cases on July 18, 2016.

Mumps RT-PCR testing was made available through the ISDHL. IgM testing was only offered through commercial laboratories. A protocol was developed to assist providers in ordering the right testing according to the time elapsed from symptom onset, collecting the correct specimens, and obtaining preauthorization for testing at ISHDL. Preauthorization required consultation with an ISDH epidemiologist to ensure patients with suspected mumps met clinical and epidemiologic criteria for testing and to ascertain exposure information to prioritize testing of specimens from patients without epidemiologic links to other cases or suspected cases in new settings. Odds ratios and comparison of proportions chi-squared tests were calculated to evaluate the impact of specimen collection timing and dissemination of testing guidance on specimen positivity. A subset of RT-PCR–positive specimens was sent to CDC’s Viral Vaccine Preventable Diseases Branch for genotyping.

**FIGURE 1:**
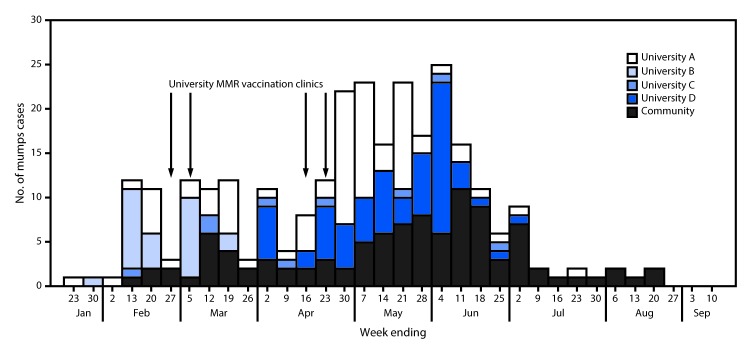
Number of confirmed (N = 237) and probable (N = 44) mumps cases associated with outbreaks at four universities, by week of onset and dates of MMR vaccination clinics — Indiana, January–September 2016 **Abbreviation:** MMR = measles, mumps, and rubella vaccine.

A total of 281 mumps cases (237 laboratory confirmed and 44 probable) were identified in all four outbreaks from January to September 2016. Among these cases, 179 (63.7%) occurred in university students or staff members (university-affiliated cases) and 102 (36.3%) in community members not affiliated with any of the universities (community cases) (Figure 1). Epidemiologic links to university cases were only identified in 25.5% of community cases. Signs and symptoms experienced by patients included parotitis (276, 98.2%), fever (109, 38.8%), headache (74, 26.3%), earache (60, 21.4%), jaw pain (16, 5.7%), malaise/body aches (11, 3.9%), and sore throat (10, 3.6%). Complications from mumps were infrequent, with one report of meningitis and five reports of orchitis.

Receipt of 2 doses of MMR was documented for 152 (84.9%) of 179 university-affiliated cases and 53 (52.0%) of 102 community cases; 11 (3.9%) of the 281 cases had documentation of a positive immunoglobulin G titer. Twelve cases (4.3%) had documentation of ≥3 doses of MMR administered >4 weeks before parotid swelling onset. In six cases in which complications occurred, the persons had each received 2 doses of MMR. Seven vaccination clinics were held across three schools, and 5,273 doses of MMR were administered, most (3,106; 59%) at highly attended clinics at university B. Based on high 2-dose MMR coverage at each university, many of these doses likely were third doses.

**FIGURE 2:**
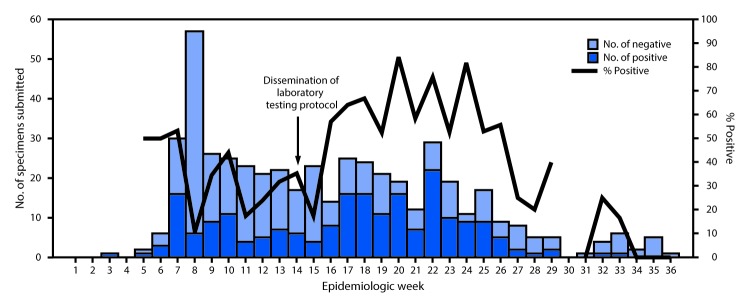
Number and percentage of specimens testing positive for mumps by reverse transcription–polymerase chain reaction, by week — Indiana State Department of Health Laboratories, 2016

ISDHL tested specimens from 490 suspected cases for confirmation by RT-PCR, 209 (42.6%) of which were positive. Among 407 cases of suspected mumps for which RT-PCR results and onset dates were available, 53.1% (146/275) of specimens collected within 2 days of parotitis onset were positive; this decreased slightly to 47.7% (63/132) for specimens collected ≥3 days after parotitis onset, and the change was not statistically significant (Table). Among 63 cases for which IgM results and onset dates were available, 34.3% (11/32) of specimens collected within 2 days of parotitis onset were positive; the rate of positivity increased to 61.3% (19/31) for specimens collected ≥3 days after parotitis onset (p<0.05). Among 18 cases for which specimens were collected within 5 days of parotitis onset and a RT-PCR test was positive, six had results that were IgM positive. Persons in 16 of these cases had received 2 MMR doses, and those in two cases had received a single dose. Weekly percent positivity of specimens submitted to ISDHL increased significantly from an average of 25.8% in the weeks before dissemination of the laboratory testing protocol to an average of 37.8% (p = 0.005) in the weeks after dissemination (Figure 2). CDC provided genotyping for 142 specimens; 140 (98.6%) were type G (the most common genotype circulating in the United States), and two were unable to be genotyped.

**TABLE:** Positivity of patient specimens for mumps, by testing method and time from symptom onset to specimen collection — Indiana, 2016

Time from onset to specimen collection	Result no. (%)		OR (95% CI)^†^
	Positive	Negative/Indeterminate*	
**RT-PCR**			
0–2 days	146 (53.1)	129 (46.9)	0.81 (0.53–1.22)
≥3 days	63 (47.7)	69 (52.3)	
**IgM**			
0–2 days	11 (34.3)	21 (65.6)	3.02 (1.08–8.44)^§^
≥3 days	19 (61.3)	12 (38.7)	

**Abbreviations:** CI = confidence interval; IgM = immunoglobulin M; OR = odds ratio; RT-PCR = reverse transcription–polymerase chain reaction.

* RT-PCR values were considered indeterminate if replicates were discordant on two separate runs. Three specimens were ruled indeterminate.

^†^ ORs and 95% CIs were calculated for test results relative to time from symptom onset to specimen collection, with specimen collection ≥3 days after symptom onset as the reference.

^§^ Significant at p<0.05 level.

## Public Health Response

Cases were classified according to the Council of State and Territorial Epidemiologists case definition for mumps (*4*), and a mumps outbreak was defined as three or more cases linked by place and time. Cases were considered infectious from 2 days before until 5 days after onset of parotitis. Control measures included isolation recommendations for persons with suspected infections, dissemination of educational materials on case finding, verification of vaccination status for persons and their close contacts in all cases, and MMR vaccination clinics at three of the four universities. Because recent studies on third-dose vaccine effectiveness were limited and had varying results (*5*,*6*), and because there was no formal ACIP recommendation regarding use of a third dose of MMR for persons affected in an outbreak at the time, no university specifically recommended a third dose of MMR to students. However, in addition to recommending to students that they attend clinics for catch-up doses of MMR, students were advised that they could receive vaccine if previous MMR vaccination documentation was unavailable or if an additional dose was desired.

Current immunization policies in Indiana require universities to collect immunization information from matriculating students at certain institutions, but guidance on record format and verification is limited (*7*). Each university had different documentation requirements for immunization records. Only two universities (B and D) required documentation of dose and month/day/year administration date, and only university B required provider verification of records.

Although isolation through 5 days after parotid swelling onset was recommended for all patients and exclusion from classes, work, or public gatherings was recommended for contacts without presumptive evidence of immunity, only university B was able to successfully ensure both isolation and exclusion by requiring either off-campus isolation or exclusion at home and providing alternative living arrangements for students who could not isolate or self-exclude off-campus Because most cases were occurring in fully vaccinated persons for whom no exclusion would be recommended by susceptibility-based exclusion policies, the benefit of enforcement was questioned, and it was difficult to garner buy-in to expend already limited personnel resources on enforcing these policies. Affected persons and contacts at universities A and C would have needed to acquire appropriate documentation of immunization from family or providers. Because of time-related difficulties in doing this, only close contacts were required to provide presumptive evidence of immunity for determining if exclusion was needed. At all universities, students without presumptive evidence of immunity were offered the option of receiving a dose of MMR and returning to campus.

## Discussion

Mumps is an acute viral illness characterized by parotid gland swelling that can result in more serious complications such as orchitis and encephalitis. A substantial increase in the number of mumps outbreaks and outbreak-associated cases has occurred in the United States since late 2014 (*8*). Four large university mumps outbreaks with considerable community spread occurred in Indiana in 2016, contributing to the 6,366 mumps cases reported nationwide in 2016, the highest number of cases in a decade. In Indiana, epidemiologic links to the university outbreaks or to other cases could not be identified for many community cases. This might indicate gaps in current case finding and linkage methods, asymptomatic transmission, or underreporting of mumps cases during nonoutbreak periods.

Laboratory testing is an important component of confirming mumps cases and outbreaks. Availability of a detailed outbreak-specific testing protocol possibly improved the overall positivity rate of specimens tested at ISDHL during the course of these outbreaks. Detection of mumps virus by RT-PCR was higher among specimens collected ≤2 days from parotid swelling onset, supporting previous findings of higher rates of positivity within 3 days of parotitis onset (*6*,*9*,*10*). Results of serologic testing support concerns regarding poor sensitivity of routine diagnostic commercial IgM testing in vaccinated persons in low-incidence settings (*10*).

The occurrence of these outbreaks highlights the need for immunization documentation requirements at institutions of higher education to be standardized and consistent with ACIP and state recommendations for documentation of presumptive evidence of immunity (*2*). As a result of this investigation, both universities A and C implemented requirements for collecting provider-verified month/day/year immunization records for all matriculating full-time students beginning in fall 2017. Although policies on exposed contact exclusion that are dependent on vaccination status can ease some difficulties in outbreak management by quickly identifying persons without evidence of immunity, these policies may be insufficient for outbreak control at institutions of higher education with high 2-dose MMR coverage (i.e., most persons and contacts are fully vaccinated). A recent ACIP recommendation states that persons previously vaccinated with 2 doses of MMR who are determined by health departments to belong to groups or populations at increased risk during a mumps outbreak be given a third dose of MMR to improve individual protection (*3*). Conducting vaccination clinics to provide these doses could allow multiple individuals to be vaccinated at once at low or no cost to students and staff members and provide an opportunity for health department personnel to educate individuals in the outbreak setting on signs and symptoms of mumps and ways to avoid infection. If outbreak management in populations with high 2-dose coverage continues to be a challenge for public health authorities despite the recommendation for use of a third dose of mumps-containing vaccine during outbreaks, reevaluation of current recommended exclusion measures could be warranted (*1*,*3*). Given the challenges in managing and controlling outbreaks in university settings, documenting and maintaining high 2-dose coverage of MMR in this setting is especially important.

**Summary**

**What is already known about this topic?**

Recently, mumps outbreaks among vaccinated persons in university settings have increased.

**What is added by this report?**

In 2016, large mumps outbreaks occurred at four Indiana universities. At some universities documentation of receipt of 2 doses of measles, mumps, and rubella vaccine (MMR) was not available and required substantial personnel time to verify. Implementation of policies for excluding susceptible persons from classes and other group settings was also difficult.

**What are the implications for public health practice?**

Outbreak-specific laboratory testing guidance to partners, standardized vaccination documentation, and evaluation of exclusion policies could aid outbreak management. The Advisory Committee on Immunization Practices currently recommends a third dose of MMR for persons at increased risk during a mumps outbreak.

## Footnotes

* The range of the incubation period for mumps virus is 12–25 days after exposure (https://www.cdc.gov/mmwr/preview/mmwrhtml/rr6204a1.htm). Two incubation periods were calculated using the maximum of the range, totaling 50 days from the final date of exposure of a university-affiliated case.

† Presumptive evidence of immunity to mumps includes any of the following: documentation of age-appropriate vaccination with a live mumps virus–containing vaccine, laboratory evidence of mumps immunity, laboratory confirmation of disease, or birth before 1957.
